# IRF4 Is a Suppressor of c-Myc Induced B Cell Leukemia

**DOI:** 10.1371/journal.pone.0022628

**Published:** 2011-07-27

**Authors:** Simanta Pathak, Shibin Ma, Long Trinh, James Eudy, Kay-Uwe Wagner, Shantaram S. Joshi, Runqing Lu

**Affiliations:** 1 Department of Genetics, Cell Biology and Anatomy, University of Nebraska Medical Center, Omaha, Nebraska, United States of America; 2 Eppley Institute for Research in Cancer and Allied Diseases, University of Nebraska Medical Center, Omaha, Nebraska, United States of America; The University of Birmingham, United Kingdom

## Abstract

Interferon regulatory factor 4 (IRF4) is a critical transcriptional regulator in B cell development and function. We have previously shown that IRF4, together with IRF8, orchestrates pre-B cell development by limiting pre-B cell expansion and by promoting pre-B cell differentiation. Here, we report that IRF4 suppresses c-Myc induced leukemia in EμMyc mice. Our results show that c-Myc induced leukemia was greatly accelerated in the IRF4 heterozygous mice (IRF4^+/−^Myc); the average age of mortality in the IRF4^+/−^Myc mice was only 7 to 8 weeks but was 20 weeks in the control mice. Our results show that IRF4^+/−^Myc leukemic cells were derived from large pre-B cells and were hyperproliferative and resistant to apoptosis. Further analysis revealed that the majority of IRF4^+/−^Myc leukemic cells inactivated the wild-type IRF4 allele and contained defects in Arf-p53 tumor suppressor pathway. p27^kip^ is part of the molecular circuitry that controls pre-B cell expansion. Our results show that expression of p27^kip^ was lost in the IRF4^+/−^Myc leukemic cells and reconstitution of IRF4 expression in those cells induced p27^kip^ and inhibited their expansion. Thus, IRF4 functions as a classical tumor suppressor to inhibit c-Myc induced B cell leukemia in EμMyc mice.

## Introduction

B cell development features a sequential rearrangement of immunoglobulin heavy and light chain loci and expression of distinct cell surface markers [1]. After productive heavy chain rearragment at the pro-B stage, the newly synthesized heavy chain pairs with surrogate light chains and forms the pre-B cell receptor (pre-BCR). Pre-B cells consist of two distinct subsets: large pre-B and small pre-B cells. Large pre-B cells are cycling cells expressing pre-BCR whereas small pre-B cells are quiescent cells following cell cycle exit. Pre-B cells expansion and the subsequent transition from large pre-B to small pre-B cells are tightly regulated during B cell development and is dependent on signals from the pre-BCR and IL-7 receptor [2]. Disruption of this coordinated developmental process can lead to abnormal B cells development and transformation. Indeed, acute lymphoblast leukemia (ALL) is often derived from pre-B cells that exhibit defects in proliferation and differentiation [3].

Interferon regulatory factor 4 (IRF4), is expressed predominantly in the immune system and plays an important role in its development and function [4]. IRF4, together with IRF8, are critical for the pre-B cell development. In the absence of IRF4 and IRF8, B cell development is blocked at the large pre-B stage [5]. We have shown that IRF4 limits pre-B cell expansion by inducing Ikaros and Aiolos which in turn directly suppress c-Myc expression [6], [7]. In addition, IRF4 is critical for light chain rearrangement and receptor editing [8], [9], [10]. Beside its role at the pre-B stage, IRF4 is also required for mature B cell function. It has been shown that mice lacking IRF4 (IRF4^−/−^) fail to generate plasma cells and are defective in response to T cell dependent and independent antigens [11]. Recent studies have further shown that IRF4 is critical for the class-switch recombination by inducing activation induced deaminase (AID) and for germinal center reaction by downregulating Bcl6 [12], [13], [14]. IRF4 has been found to induce c-Myc expression in multiple myeloma cells and is critical for their survival and expansion [15]. Finally, IRF4 can induce the expression of Fas apoptosis inhibitory molecule (FAIM) to regulate mature B cell survival and apoptosis [16].

Given its role as a critical transcriptional regulator that limits pre-B cell expansion and promotes pre-B cell differentiation, it is reasonable to assume that IRF4 may function as a tumor suppressor against pre-B cell transformation. Indeed, a previous study has shown that IRF4 functions as a tumor suppressor to inhibit BCR/ABL oncogene induced B cell acute lymphoblastic leukemia (B-ALL) [17]. In addition, mice deficient for both IRF4 and IRF8 develop lymphoblastic leukemia [18]. Although IRF4 can suppress BCR/ABL induced B cell transformation, the molecular mechanism by which IRF4 exerts its function remains unclear. In this report, we assessed the role of IRF4 in c-Myc oncogene induced B cell transformation by breeding IRF4 deficient mice with EμMyc transgenic mice. In the EμMyc mice, the expression of c-Myc oncogene is driven by immunoglobulin heavy chain enhancers and is predominantly found in the B cells. EμMyc transgenic mice mainly develop two types of leukemia/lymphoma: pro/pre-B derived and mature B cell derived and the majority of the EμMyc mice succumb to disease within 5 to 6 months of age [19]. It has been shown that the leukemogenesis of EμMyc mice can be modulated by oncogenes and tumor suppressor genes and thus, EμMyc mice have been widely used as an animal model to assess the role of potential oncogenes or tumor suppressor genes in B cell transformation [20], [21], [22], [23].

In this report, we show that c-Myc induced leukemia was greatly accelerated in the IRF4 heterozygous mutant mice. Moreover, we provided evidence that IRF4 functions as a classical tumor suppressor gene to inhibit c-Myc induced leukemogenesis. Our results further revealed that deficiency of IRF4 accelerated the loss of p27^kip^ in the EμMyc mice and reconstitution of IRF4 expression in leukemic cells restored p27^kip^ expression in leukemic cells and inhibited their proliferation *in vivo*.

## Results

### C-Myc induced leukemia was accelerated in the IRF4^+/−^ Myc mice

**Figure 1:**
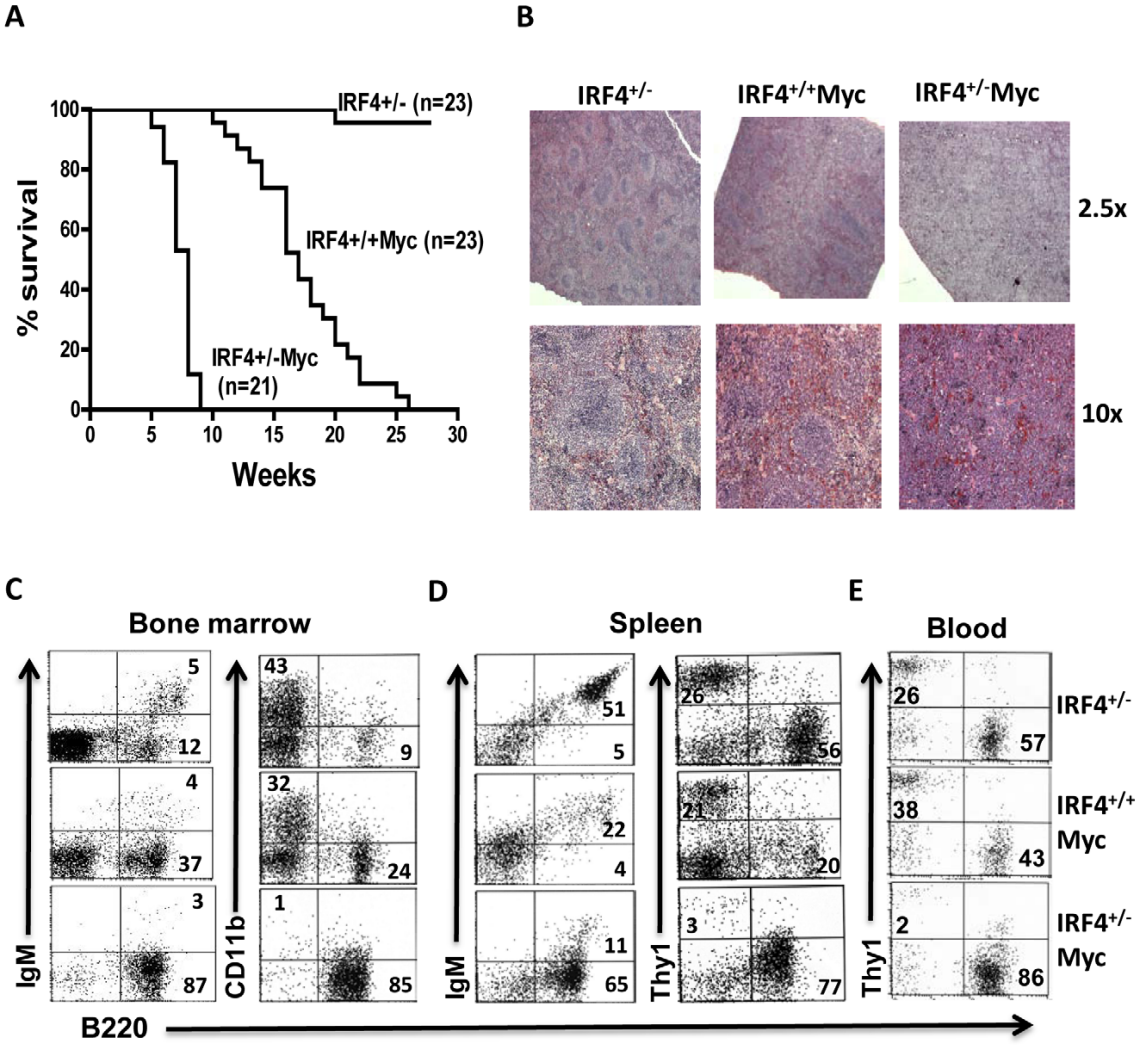
c-Myc induced leukemia was accelerated in the IRF4^+/−^ Myc mice. A). Kaplan-Meier survival curve of IRF4^+/−^, IRF4^+/+^Myc and IRF4^+/−^Myc mice. The genotypes and numbers of mice in each group are indicated on the plot. P value for pair-wise comparison using log rank test between IRF4^+/+^Myc and IRF4^+/−^Myc is p<0.0001. Graphpad PRISM 5.03 (Graphpad Software Inc) was used to plot the survival curve and calculate p value. B) Spleens were isolated from IRF4^+/−^, IRF4^+/+^Myc and IRF4^+/−^Myc mice and paraffin-embedded for H&E staining analysis. The stained tissues were examined under both low (2.5×) and high (10×) magnifications. C/D/E). Cells were isolated from the bone marrow (C), spleen (D) and blood (E) of six-week old IRF4^+/−^, IRF4^+/+^Myc and IRF4^+/−^Myc mice, stained with indicated antibodies and analyzed by FACS. Numbers indicated the percentages of cells in the respective quadrant. CD11b+ myeloid cells in the bone marrow were examined under a live cell gate while B and T cells were examined under a lymphocyte gate. The data shown are representative of at least three independent experiments.

We did not observe a significant increase in tumor formation in IRF4 deficient mice, indicating that deficiency for IRF4 alone isn't sufficient for tumor development. Here, we wanted to examine whether deficiency of IRF4 synergizes with the c-Myc oncogene to induce B cell leukemia and lymphoma. We crossed IRF4^−/−^ mice with the EμMyc mice and generated IRF4 heterozygous mutant mice expressing EμMyc transgene (IRF4^+/−^Myc). To our surprise, IRF4^+/−^Myc mice showed a dramatically accelerated mortality with a median age of 7 to 8 weeks (Fig. 1A). In contrast, the median age of mortality for IRF4^+/+^Myc mice is 20 weeks (Fig. 1A). By five weeks of age, IRF4^+/−^Myc mice had already massively enlarged spleens, while this was not evident in the IRF4^+/+^Myc mice of the same age (Figure S1A). H&E staining of isolated spleens further shows that while the demarcation of white and red pulps is clearly discernible in IRF4^+/−^ and IRF4^+/+^Myc mice, it disappeared in the IRF4^+/−^Myc mice due to the expansion and infiltration of leukemic cells.

FACS analysis of cells in the bone marrow and spleen further show that there was a massive expansion of B220+ B cells in the IRF4^+/−^Myc mice (Fig. 1C/D and Tab. 1/2). Compared to IRF4^+/+^Myc mice, the absolute numbers of B220+ B cells was found to be 3-fold higher in bone marrow and 10-fold higher in spleen. While the absolute numbers of B220+IgM+ B cells in bone marrow and spleen were comparable between IRF4^+/+^Myc and IRF4^+/−^Myc mice, the absolute numbers of B220+IgM− B cells were dramatically increased in the IRF4^+/−^Myc mice (Fig. 1C/D and Tab 1/2).The massive expansion of leukemic cells also severely affected the development of other blood lineages. As shown in Fig. 1C, the percentages of CD11b+ myeloid cells in bone marrow decreased from 32% in the IRF4^+/+^Myc mice to only 1% in the IRF4^+/−^Myc mice. In spleen, the percentage of Thy1+ T cells decreased from 21% in IRF4^+/+^Myc mice to only 3% in the IRF4^+/−^Myc mice. The expansion and infiltration of leukemic cells also caused severe erosion and thinning of the femus and tibia. In addition, the B cell population (mainly B220+IgM−) was also increased in the blood of IRF4^+/−^Myc mice (Fig. 1E and data not shown). The leukemic cells also infiltrated the lymph node, Thymus and liver, resulting in mild to moderate enlargement of those organs. Two additional sets of independent analysis can be found in Figure S1. Collectively, our results indicate that the IRF4^+/−^Myc mice developed leukemia that originated from B cell progenitors.

**Table 1:** Absolute number of B220+, B220+IgM− and B220+IgM+ B cells in bone marrow of IRF4+/+, IRF4+/−, IRF4+/+Myc and IRF4+/−Myc mice (×10^6^).

Genotype	B220+	B220+IgM−	B220+IgM+
IRF4+/+	3.8±2.1	2.5±1.4	1.3±0.6
IRF4+/−	3.1±0.7	1.9±0.6	1.0±0.2
IRF4+/+Myc	6.1±2.3	5.1±2.7	1.0±0.1
IRF4+/−Myc	19.5±6.4**	18.0±5.7**	1.3±0.4

Total number of B220+, B220+IgM− and B220+IgM+ B cells in mice of different genotypes under study. Cell were isolated from bone marrow of five-week- old IRF4+/+, IRF4+/− IRF4+/+Myc and IRF4+/−Myc mice. The cells were stained with antibodies against B220 and IgM and analyzed by FACS. The numbers are the averages and standard deviations of the results for a total of five mice in each group.

**p<0.01 (compared to their counterparts in the IRF4+/+Myc mice).

**Table 2:** Absolute number of B220+, B220+IgM− and B220+IgM+ B cells in spleen of IRF4+/+, IRF4+/−, IRF4+/+Myc and IRF4+/−Myc mice (×10^6^).

Genotype	B220+	B220+IgM−	B220+IgM+
IRF4+/+	29.7±13.5	0.9±0.4	29.6±12.5
IRF4+/−	15.0±6.2	0.5±0.4	14.7±5.8
IRF4+/+Myc	14.7±7.8	0.6±0.3	14.3±8.0
IRF4+/−Myc	141.0±48.0**	128.0±40.4**	12.7±7.8

Total number of B220+, B220+IgM− and B220+IgM+ B cells in mice of different genotypes under study. Cell were isolated from spleen of five-week- old IRF4+/+, IRF4+/− IRF4+/+Myc and IRF4+/−Myc mice. The cells were stained with antibodies against B220 and IgM and analyzed by FACS. The numbers are the averages and standard deviations of the results for a total of five mice in each group.

**p<0.01 (compared to their counterparts in the IRF4+/+Myc mice).

### IRF4^+/−^Myc leukemic cells were derived from large pre-B cells and were transplantable

**Figure 2:**
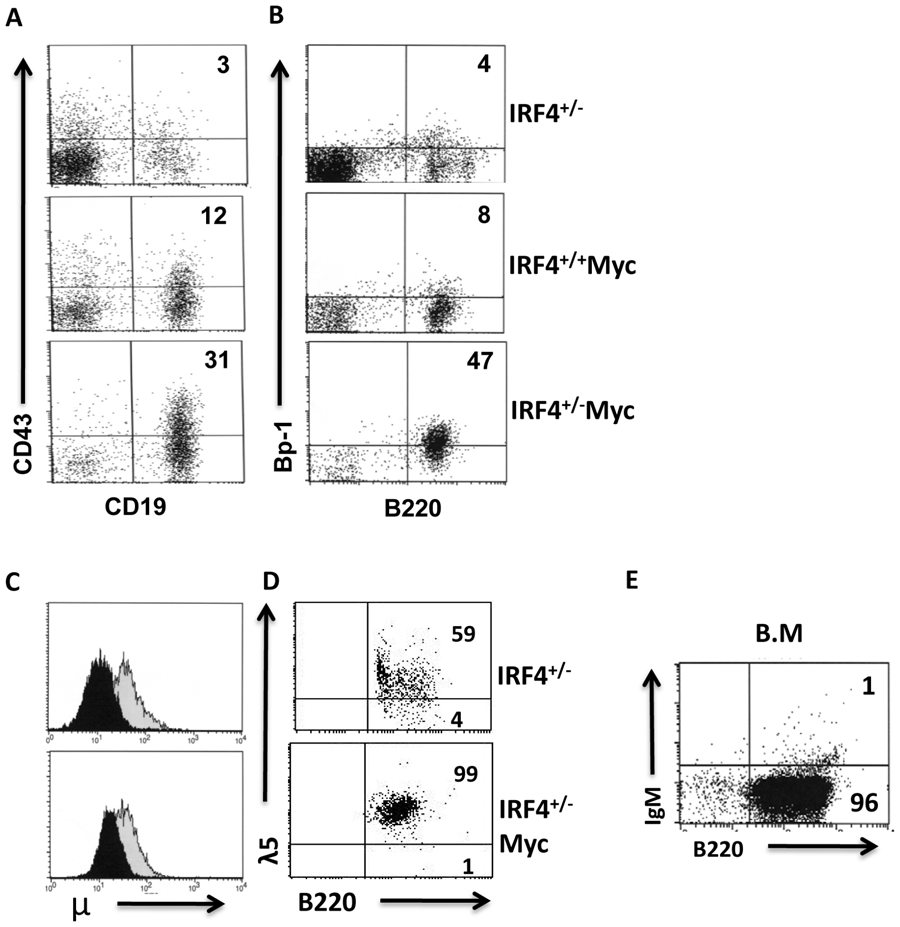
IRF4^+/−^Myc leukemic cells were derived from large pre-B cells and were transplantable. A/B/) Bone marrow cells were isolated from six-week old IRF4^+/−^, IRF4^+/+^Myc and IRF4^+/−^Myc mice. The cells were stained with antibodies against CD43, Bp-1, CD19 and B220. The stained cells were analyzed by FACS. Numbers are percentages of cells in the respective quadrant. C) IRF4^+/−^Myc leukemic cells expressed intracellular μ. The bone marrow cells isolated above were stained with antibodies against B220 and CD43. After fixation and permeablization, the expression of intracellular heavy chain μ was detected with an anti-IgM antibody. The isotype IgG1 antibody staining was uses as a control for non-specific binding. The dark area (control IgG1) and light area (anti-IgM). D) IRF4^+/−^Myc cells expressed surrogate light chain λ5. Bone marrow cells isolated from IRF4+/− and IRF4^+/−^Myc mice were cultured in presence of IL-7 for two days. The expression of surrogate light chain λ5 was detected by FACS. E) Bone marrow cells were isolated from IRF4+/−Myc mice and transplanted into the non-irradiated syngenic host mice at 1×10^6^ cells per mice. A total of 10 host mice were used. The host mice were analyzed by FACS after 5 to 10 weeks. A representative bone marrow analysis was shown.

To determine the identity of B220+IgM− cells in the IRF4^+/−^Myc mice, we stained the cells with a panel of cell surface as well as intracellular markers such as CD43, Bp-1, intracellular mu and surrogate light chain λ5. Pre-B cells express Bp-1 but do not express CD43. The heavy chain protein mu (μ) can be detected intracellularly in the pre-B but not the pro-B cells. λ5 is a component of pre-B cell receptor found only on the cell surface of pre-B cells. FACS analysis showed that B220+IgM− leukemic cells in the IRF4^+/−^Myc mice did not express CD43 but express a high level of Bp-1, intracellular μ and surrogate light chain λ5 (Fig. 2A/B/C/D). Collectively, these results indicate that the B220+IgM− leukemic cells were derived from the large pre-B cells. To determine if the leukemic cells derived from IRF4^+/−^Myc mice are transplantable, we injected IRF4^+/−^Myc bone marrow cells into non-irradiated wild type syngenic host mice. All ten recipient mice developed leukemia similar to IRF4^+/−^Myc mice (Fig. 2E) and died within two month of transplantation. Two additional sets of independent analysis can be found in Figure S2. In summary, IRF4^+/−^Myc mice develop pre-B cells derived leukemia that is transplantable in the syngenic mice.

### The B220+IgM− cells in IRF4^+/−^Myc mice exhibited enhanced proliferation and reduced apoptosis

**Figure 3:**
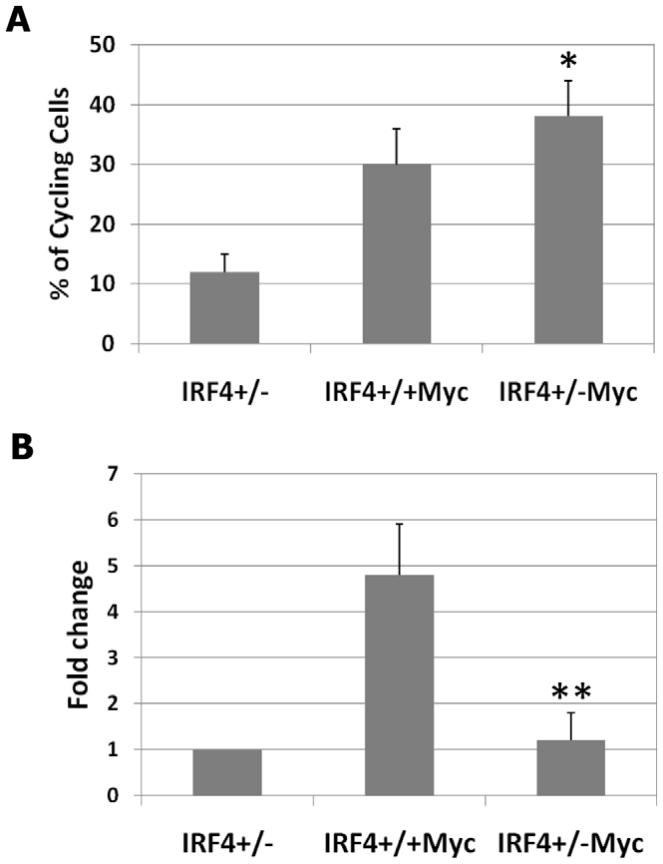
B220+IgM− cells in IRF4^+/−^Myc mice exhibited enhanced proliferation and reduced apoptosis. Bone marrow cells were isolated from four-week old IRF4^+/−^, IRF4^+/+^Myc and IRF4^+/−^Myc mice. The cells were stained with antibodies against B220 an IgM. A) To analyze cell cycle status, the stained cells were incubated with Hoechst dye (10 µg/ml) and analyze by LSR II Flow Cytometer. The percentages of cycling cells among B220+IgM− population in different groups were shown. B) To detect apoptotic cells, the stained cells were fixed and permeablized. The percentages of apoptotic cells among B220+IgM− population were determined with a TUNEL kit and plotted as fold changes over IRF4^+/−^ control mice. The results are average and standard deviation of the values obtained from six independent experiments. * p<0.05; ** p<0.01.

We wanted to identify the molecular defects that led to the accelerated leukemogenesis in the IRF4^+/−^Myc mice. To this end, we examined the survival and proliferation of B220+IgM− cells in the bone marrow of 4-weeks old IRF4^+/−^Myc mice prior to the development of symptoms. At this age, although there was expansion of B220+IgM− cells in the bone marrow and spleen of IRF4^+/−^Myc mice, there were few B220+IgM− cells in the blood (data not shown). Bone marrow cells were isolated from IRF4^+/−^, IRF4^+/+^Myc and IRF4^+/−^Myc mice. After surface staining, the cell cycle status of B220+IgM− cells was examined with Hoechst dye as previously described [6], [7]. Compared to the IRF4^+/+^Myc mice, B220+IgM− cells in the IRF4^+/−^ Myc mice were hyperproliferative as 38±8% of B220+IgM− cells were cycling (in S and G2/M phases) in the IRF4^+/−^Myc mice compared to 30±6% in the IRF4^+/+^Myc mice (Fig. 3A). BrdU pulse-labeling analysis further revealed that the B220+IgM− cells in IRF4^+/−^ Myc mice were cycling faster than their counterparts in the IRF4^+/+^Myc mice (data not shown). TUNEL analysis further revealed that 4.8±1.1% of B220+IgM− cells in the IRF4^+/+^Myc mice were apoptotic whereas only 1.2±0.6% of them in the IRF4^+/−^Myc mice underwent apoptosis (Fig. 3B). Collectively, these results show that the expanded B220+IgM− cells in the IRF4^+/−^Myc mice are hyperproliferative and resistant to apoptosis.

### Loss the expression of p27^kip^ and disruption of the Arf-p53 pathway in the IRF4^+/−^Myc leukemic cells

**Figure 4:**
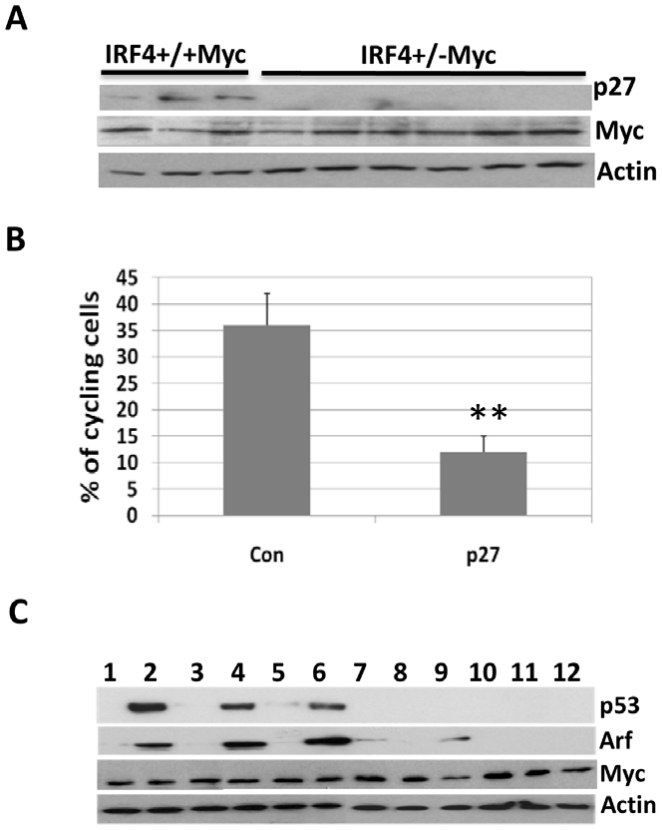
loss the expression of p27^kip^ and disruption of Arf-p53 pathway in IRF4^+/−^Myc leukemic cells. A) Bone marrow cells were isolated from four-week old IRF4^+/+^Myc (n = 3) and IRF4^+/−^Myc mice (n = 6). The B220+IgM− population were isolated via sorting and were lysed. Western blot analysis was carried out with antibodies against p27, Myc and β-actin. B) IRF4^+/−^Myc leukemic cells were cultivated in RPMI-1640 plus 5% FBS in the presence of IL7 (5 ng/ml). The cells were infected with retrovirus expressing either MigR1 (control) or MigR1-p27 (p27). Two days after infection, the infected cells were incubated with Hoechst dye and analyzed by LSR II Flow Cytometer. The percentages of cycling cells in control and p27 infected cells were shown. The results are average and standard deviation of the values obtained from three independent experiments. ** p<0.01 C) leukemic cells were isolated from twelve sick IRF4^+/−^Myc mice and lysed for Western blot analysis. The antibodies against p53, Arf, Myc and β-actin were used.

P27^kip^ is a cell cycle inhibitor, and loss of p27^Kip^ expression has been shown to accelerate c-Myc induced leukemia and lymphoma [23]. Moreover, our previous study has shown that p27^kip^ is part of molecular circuitry that is responsible for shutting down pre-B cell proliferation [6]. Here, we further examined the expression of p27^kip^ in the B220+IgM− cells described in Fig. 3A. Although p27^kip^ expression was readily detectable in all three IRF4^+/+^Myc cells, its expression was lost in all six IRF4^+/−^ Myc cells (Fig. 4A). Importantly, expression of c-Myc was comparable between IRF4^+/+^Myc and IRF4^+/−^Myc cells (Fig. 4A). To further assess the role of p27^kip^ in the proliferation of IRF4^+/−^Myc leukemic cells, we transduced cultured IRF4^+/−^Myc leukemic cells with p27^kip^ expression plasmid. As shown in Fig. 4B, restoring the expression of p27^kip^ dramatically inhibited the proliferation of IRF4^+/−^Myc leukemic cells. The percentage of cycling cells decreased from 35% in the control to 12% in the p27^kip^ transduced cells. Collectively, these results suggest that defective expression of p27^kip^ contributes to the hyperproliferative index of IRF4^+/−^Myc leukemic cells.

The development of leukemia and lymphoma in EμMyc mice is dependent on the disruption of Arf-p53 tumor suppressor pathway [24], [25]. It has been shown that p53 is frequently mutated in EμMyc tumor [24]. The mutant form of p53 is often overexpressed and functions as a dominant negative mutant. To determine if Arf-p53 pathway is disrupted in the IRF4^+/−^Myc leukemic cells, we measured the expression of p53, Arf and c-Myc in IRF4^+/−^ leukemic cells isolated from twelve different diseased mice. Our results show that p53 was overexpressed in three IRF4^+/−^Myc leukemic cells (Fig. 4C). Further sequence analysis shows that all three cells contained a p53 misense mutation R270C, which corresponds with a mutation hotspot of p53 found in human tumors [26]. Expression of Arf is transcriptionally suppressed by p53 and the loss of p53 function often lead to overexpression of Arf in EμMyc tumor cells. Indeed, Arf was overexpressed in IRF4^+/−^Myc leukemic cells expressing mutant p53 (Fig. 4C). Overall, three out of twelve IRF4^+/−^Myc clones expressed mutant p53, a frequency that is similar to what has been reported in wild type EμMyc mice [24].

### IRF4 functions as a tumor suppressor in c-Myc induced leukemia

**Figure 5:**
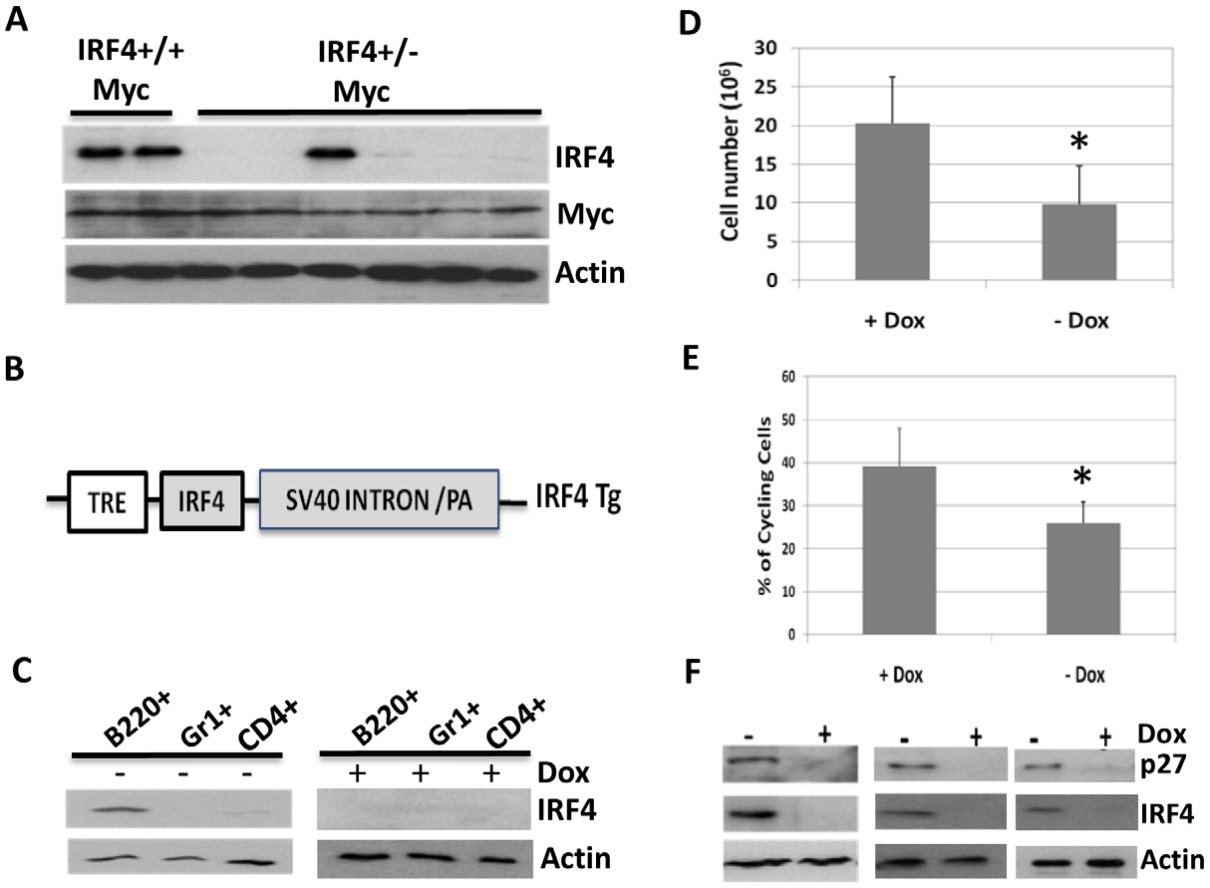
IRF4 functions as a tumor suppressor in c-Myc induced leukemia. A) IRF4+/−Myc leukemic cells lost the wild type IRF4 allele. B220+IgM− cells were isolated from bone marrow of sick IRF4^+/−^Myc mice (n = 6) and two IRF4^+/+^Myc littermate control mice. The B220+IgM− cells were lysed for Western blot analysis with antibodies against IRF4, Myc and β-actin. B) Map of IRF4 transgenic construct. The diagram depicts the constructs of IRF4 transgenic construct including tetracycline response element (TRE) and SV40 intron and ployA tail. C) IRF4 transgene was predominantly expressed in B cells. IRF4Tg and MMTV-tTA single transgenic mice were crossed to generate double transgenic mice (Tg-tTA). B cells, T cells and granulocytes were isolated from Tg-tTA double transgenic mice one week after Dox removal. The cells isolated from Tg-tTA mice fed with Dox water were lysed and used as control. The expression of the IRF4 transgene was detected by Western Blot using an anti-HA antibody. D/E) Expression of IRF4 transgene inhibited the expansion of IRF4^+/−^Myc leukemic cells. The Tg-tTA transgenic mice crossed with IRF4 deficient mice and EμMyc mice to generate IRF4^+/−^ mice that were hemizygous for Tg-tTA and EμMyc transgene (IRF4^+/−^MycTg-tTA). At five weeks of age, IRF4^+/−^MycTg-tTA mice were either switched to regular water to induce transgene expression or continuously fed with dox water. After another three weeks, the mice were sacrificed and analyzed by FACS. Numbers of B220+IgM− cells in the bone marrow of IRF4^+/−^MycTg-tTA mice were counted (D). In addition, percentages of cycling cells among B220+IgM− population were also analyzed using method described above (E). The results were averages and standard deviations of six independent experiments. *p<0.05. F) Expression of IRF4 transgene induced the expression of p27 in IRF4^+/−^Myc leukemic cells. B220+IgM− cells were isolated from three pairs of IRF4^+/−^MycTg-tTA mice in presence or absence of Dox and lysed for Western blot analysis. The effect of IRF4 transgene expression on p27 expression was examined.

The finding that c-Myc induced leukemia was dramatically accelerated in the IRF4^+/−^ mice indicates that IRF4 functions as a tumor suppressor in c-Myc induced leukemia. During tumorigenesis, loss of one copy of tumor suppressor genes is often followed by inactivation of the second allele. Indeed, in IRF4^+/−^Myc leukemic cells, the expression of IRF4 is loss in five out of six IRF4^+/−^Myc leukemic cells examined (Fig. 5A). Further sequence analysis of mRNA and DNA did not reveal deletion or mutations of IRF4 gene, suggesting that loss of IRF4 expression in the IRF4^+/−^Myc leukemic cells could be mediated by an epigenetic event or by the loss of essential transcriptional regulators. In contrast, the expression of c-Myc was not significantly affected in the IRF4^+/−^Myc leukemic cells.

To further examine the effect of IRF4 on c-Myc induced leukemogenesis, we developed a doxycycline (Dox) inducible, bitransgenic mouse model system in which the expression of IRF4 can be regulated *in vivo* by Dox. The first transgenic line MMTV-tTA was developed by Hennighausen et al, in which the expression of the tetracycline transactivator gene (tTA) is driven by the mouse mammary tumor virus LTR [27]. In this system, the tTA is active in the absence but not in the presence of Dox. Interestingly, it has been demonstrated that the tTA is expressed at a high level in the B cells of MMTV-tTA mice and thus, this model has been successfully used to induce B cell specific transgene expression [28], [29], [30], [31]. As shown in Fig. 5B, expression of the IRF4 transgene is driven by a TRE promoter which consists of a minimal CMV promoter linked to tetracycline response elements.

IRF4Tg and MMTV-tTA transgenic mice were crossed to generate double transgenic mice that are hemizygous for IRF4Tg and MMTV-tTA (Tg-tTA). The mice were initially fed with a high concentration of Dox (2 mg/ml Dox, 4% sucrose) for couple days and then switched to a low concentration to keep the transgene inactive. The expression of the IRF4 transgene was induced in mice by switching them to regular water. To examine the expression pattern of the IRF4 transgene in immune cells, we isolated B cell (B220+), granulocytes (Gr1+), and CD4+ T cells from bone marrow and spleen of Tg-tTA mice. Expression of the IRF4 transgene was detected with an anti-HA antibody. As shown in Fig. 5C, the IRF4 transgene was predominantly expressed in the B cells in the absence of Dox.

The Tg-tTA transgenic mice were subsequently crossed with IRF4 deficient mice and EμMyc mice to generate triple transgenic mice in the IRF4 heterozygous background (IRF4^+/−^MycTg-tTA). At five weeks of age, IRF4^+/−^MycTg-tTA mice were either switched to regular water to induce transgene expression or continuously fed with dox water. After another three weeks, the mice were sacrificed and analyzed. The total number of B220+IgM− B cells was dramatically reduced in the bone marrow and spleens of IRF4^+/−^MycTg-tTA mice fed with regular water (Fig. 5D and data not shown), indicating that expression of IRF4 transgene inhibited the expansion of IRF4^+/−^Myc leukemic cells. Cell cycle analysis further shows that expression of IRF4 transgene inhibited the proliferation of IRF4^+/−^Myc cells (Fig. 5E). Western blot analysis revealed that expression of p27^kip^ was elevated in the IRF4^+/−^Myc cells expressing IRF4 transgene. Collectively, these results indicate that reconstitution of IRF4 expression induces p27^kip^ and inhibits the proliferation of IRF4^+/−^Myc leukemic cells.

## Discussion

In this report, we provide evidence that IRF4 functions as a tumor suppressor in c-Myc induced B cell leukemia. Our results show that c-Myc induced leukemia was dramatically accelerated in IRF4^+/−^Myc mice. Moreover, five out of six of IRF4^+/−^Myc leukemic clones further inactivated the remaining wild type IRF4 allele, resulting in a complete loss of IRF4 expression in those cells. Our finding is consistent with a previous study which shows that IRF4 functions as tumor suppressor in BCR/ABL oncogene induced B-ALL and further demonstrates that IRF4 is capable of functioning as a tumor suppressor against a broad spectrum of oncogene insults at the pre-B stage. The accelerated leukemogenesis in the IRF4+/−Myc mice can be caused by defect in pre-B cell development. Progenitor B cells are expanded in wild type EμMyc mice. As IRF4 is a critical regulator of pre-B cell differentiation, loss of IRF4 expression can further exacerbate the defect in early B cell development in the EμMyc mice, causing further expansion of progenitor B cells pool that could serve as targets for subsequent transformation.

C-Myc induced leukemia and lymphoma is held in check by p27^kip^ which inhibit cell cycle progression and by the Arf-p53 pathway that promotes apoptosis [20], [24], [32]. However, the frequency of p53 mutation in the IRF4^+/−^Myc leukemic cells is similar to wild type EμMyc cells, suggesting that accelerated leukemogenesis in IRF4^+/−^Myc mice isn't a result of high frequency of p53 mutation. Instead, our results indicate that deficiency of IRF4 accelerates the loss of p27^kip^ in c-Myc overexpressing cells. First, the expanded B220+IgM-cells in IRF4^+/−^Myc mice lost the expression of p27^kip^ and were hyperproliferative; second, reconstitution of IRF4 expression in IRF4^+/−^Myc leukemic cells induced the expression of p27^kip^ and inhibited their proliferation. It has been shown that Ikaros can induce the expression p27^kip^ in leukemic cells [33]. As expression of Ikaros is induced by IRF4, it is possible that the induction of p27^kip^ expression could be mediated by Ikaros. It is also possible that loss of IRF4 expression somehow accelerates the loss of p27^kip^ in c-Myc overexpressing cells through mechanisms independent of Ikaros.

Pre-B cell receptor signaling has been proposed as a guardian of pre-B cell transformation by coordinating pre-B cell expansion and differentiation. IRF4 expression is dependent on pre-BCR signaling which is transduced by downstream signaling molecules such as Blnk, Btk and PLCγ2. Moreover, previous studies have identified Btk, Blnk and PLCγ2 as potential tumor suppressors against pre-B cell transformation [34], [35], [36], [37]. Interestingly, a previous study has shown that c-Myc induced leukemia/lymphoma is accelerated in PLCγ2 deficient mice (PLCγ2^−/−^Myc) [34]. Similar to IRF4, PLCγ2 is critical for pre-B cell development and in its absence, pre-B cell development is partially blocked, resulting in an expansion of pre-B cells. Interestingly, like IRF4^+/−^Myc leukemic cells, PLCγ2^−/−^Myc tumor cells lost the expression of p27^kip^ and didn't exhibit enhanced frequency of p53 mutation [34]. However, PLCγ2 does not behave like classical tumor suppressor as c-Myc induced leukemogenesis remains unaltered in the PLCγ2^+/−^Myc mice. In contrast, our results show that IRF4 behaves like a classical tumor suppressor, downstream of pre-BCR signaling, that functions to inhibit c-Myc induced leukemia.

## Materials and Methods

### Mice

IRF4 mutant mice (IRF4^−/−^) have been previously described [11]. EμMyc transgenic mice were purchased from Jackson Laboratories [19]. EμMyc transgenic mice were bred with IRF4^−/−^ mice to generate IRF4^+/−^ mice that are hemizygous for Myc transgene (IRF4^+/−^Myc). All mice were maintained under specific pathogen-free conditions. Experiments were performed according to guidelines from the National Institutes of Health and with an approved protocol from the Institutional Animal Care and Use Committee of University of Nebraska Medical Center (Permit Number: 10-015-05). The mice aged from 5 to 30 weeks were used for this study.

#### Cell culture and retroviral infection

B220+ cells were isolated from bone marrow of IRF4^+/−^Myc mice using a MACS separation column (Miltenyi Biotech). Purified cells were overlaid on top of an irradiated S17 stromal cell layer in Opti-MEM (Gibco) medium containing 5% FBS, 50 µM β-mercaptoethanol, 2 mM L-glutamine, 100 U penicillin-streptomycin and 5 ng/ml IL-7 (R&D). S17 is a bone marrow stromal cell line that can support both myeloid and B lymphocyte development. Retroviral vectors expressing p27^kip^ have been described previously [7]. Retroviral infection of cultured IRF4^+/−^Myc leukemic cells was conducted as described previously [7]. The infected cells were analyzed by FACS two days after infection.

### Fluorescence-activated cell sorter analysis (FACS), TUNEL, and cell cycle analyses

5–10 weeks old IRF4^+/+^, IRF4^+/−^, IRF4^+/+^Myc and IRF4^+/−^Myc mice were used for FACS analysis. Cells were pre-incubated with either 2% rat serum or Fc-Block (2.4G2), and stained with optimal amounts of specific antibodies, either biotinylated or directly fluorophore-conjugated. Antibodies against B220 (RA3-6B2), CD19 (ID3), Thy-1, CD43, Bp-1, IgM and λ5 were purchased from Pharmingen; FACS analysis was performed with a FACS Calibur flow cytometer. The terminal deoxynucleotidyltranferase-mediated dUTP-biotin nick end labeling (TUNEL) assay was carried out with an APO-Direct Kit (Pharmingen). B cells labeled with dUTP in the absence of terminal transferase were used as negative control. The stained cells were analyzed by FACS. Cell cycle analysis with live cells was conducted using Hoechst 33342 dye as previously described [7].

### Bone marrow transplantation

Bone marrows cells were isolated from hind limbs of the mice. The whole bone marrow cells were injected via retro-orbital sinus into nonirradiated syngenic host mice at 1×10^6^ cells per mice. B cell population in the recipient mice was analyzed by FACS after five to 10 weeks.

### Western Blot and Immunohistochemistry analysis

Isolated B cells were lysed and used for Western blot analysis. The signals were visualized using the SuperSignal West Dura HRP Detection kit (Pierce). The information for the antibodies used in this study: antibodies against IRF4, p27^kip^, Myc and β-actin (Santa Cruz); antibody against Arf (ab80, Abcam) and antibody against p53 (Ab7, Calbiochem). For immunohistochemical analysis, the spleens were fixed in formalin and paraffin-embedded. H&E staining was carried out at the tissue process core facility of the University of Nebraska Medical Center.

### Generation of an inducible IRF4 transgenic mouse

To generate Doxycline (Dox) responsive IRF4 transgenic mice, a full-length IRF4 cDNA with a HA tag at the N-terminus was inserted into the pTet-Splice vector, generating the transgenic plasmid (IRF4Tg). The expression of the IRF4 transgene is driven by a TRE promoter which consists of a minimal CMV promoter fused with a tetracycline response element. Microinjections were performed by the Transgenic Mouse Core Facility at the University of Nebraska Medical Center. Six different founder lines were established and crossed with another transgenic line which expresses tetracycline transactivator gene MMTV-tTA mice (Jackson lab). In this system, tTA becomes active in the absence but not in the presence of Dox. After crossing, all lines were found to express IRF4 in the absence of Dox and the one expressing the highest level of IRF4 was chosen for further analysis. All mice were maintained under specific pathogen-free conditions. Experiments were performed according to guidelines from the National Institutes of Health and with an approved protocol from the Institutional Animal Care and Use Committee of University of Nebraska Medical Center (Permit Number: 10-015-05). The mice aged from 5 to 30 weeks were used for this study.

## Supporting Information

Figure S1 **Two additional sets of independent experiments to show that c-Myc induced leukemia is accelerated in the IRF4^+/−^Myc mice.** A) splenomegaly in IRF4^+/−^Myc mice B) Spleens were isolated from IRF4^+/−^, IRF4^+/+^Myc and IRF4^+/−^Myc mice and paraffin-embedded for H&E staining analysis. The stained tissues were examined under both low (2.5×) and high (10×) magnifications. C/D/E). Cells were isolated from the bone marrow (C), spleen (D) and blood (E) of six-week old IRF4^+/−^, IRF4^+/+^Myc and IRF4^+/−^Myc mice, stained with indicated antibodies and analyzed by FACS. Numbers indicated the percentages of cells in the respective quadrant. CD11b+ myeloid cells in the bone marrow were examined under a live cell gate while B and T cells were examined under a lymphocyte gate. The data shown are representative of at least three independent experiments.
(TIF)

Figure S2 **Two additional sets of independent experiments to show that IRF4^+/−^Myc leukemic cells were derived from large pre-B cells and were transplantable.** A/B/) Bone marrow cells were isolated from six-week old IRF4^+/−^, IRF4^+/+^Myc and IRF4^+/−^Myc mice. The cells were stained with antibodies against CD43, Bp-1, CD19 and B220. The stained cells were analyzed by FACS. Numbers are percentages of cells in the respective quadrant. C) IRF4^+/−^Myc leukemic cells expressed intracellular μ. The bone marrow cells isolated above were stained with antibodies against B220 and CD43. After fixation and permeablization, the expression of intracellular heavy chain μ was detected with an anti-IgM antibody. The isotype IgG1 antibody staining was uses as a control for non-specific binding. The dark area (control IgG1) and light area (anti-IgM). D) IRF4^+/−^Myc cells expressed surrogate light chain λ5. Bone marrow cells isolated from IRF4+/− and IRF4^+/−^Myc mice were cultured in presence of IL-7 for two days. The expression of surrogate light chain λ5 was detected by FACS. E) Bone marrow cells were isolated from IRF4+/−Myc mice and transplanted into the non-irradiated syngenic host mice at 1×10^6^ cells per mice. A total of 10 host mice were used. The host mice were analyzed by FACS after 5 to 10 weeks. Two representative bone marrow analysis were shown.
(TIF)
