# Supplementary figures and images for: IRF4 Is a Suppressor of c-Myc Induced B Cell Leukemia
Source: PLoS One. 2011 Jul 27;6(7):e22628. doi: 10.1371/journal.pone.0022628 (PMC3144921; doi:10.1371/journal.pone.0022628)

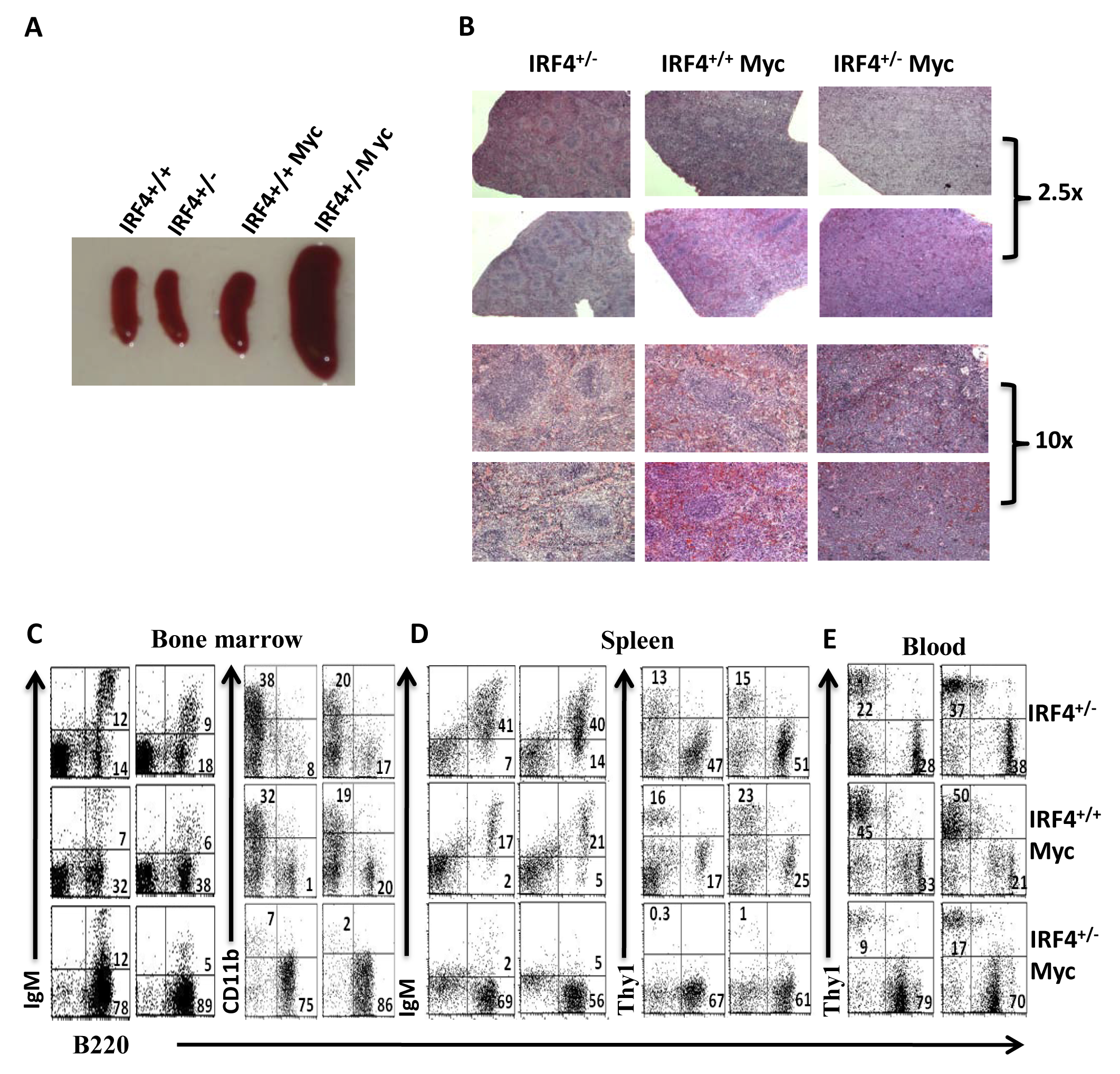

Supplement: Figure S1 — Two additional sets of independent experiments to show that c-Myc induced leukemia is accelerated in the IRF4+/−Myc mice. A) splenomegaly in IRF4+/−Myc mice B) Spleens were isolated from IRF4+/−, IRF4+/+Myc and IRF4+/−Myc mice and paraffin-embedded for H&E staining analysis. The stained tissues were examined under both low (2.5×) and high (10×) magnifications. C/D/E). Cells were isolated from the bone marrow (C), spleen (D) and blood (E) of six-week old IRF4+/−, IRF4+/+Myc and IRF4+/−Myc mice, stained with indicated antibodies and analyzed by FACS. Numbers indicated the percentages of cells in the respective quadrant. CD11b+ myeloid cells in the bone marrow were examined under a live cell gate while B and T cells were examined under a lymphocyte gate. The data shown are representative of at least three independent experiments. (TIF) [file pone.0022628.s001.tif]

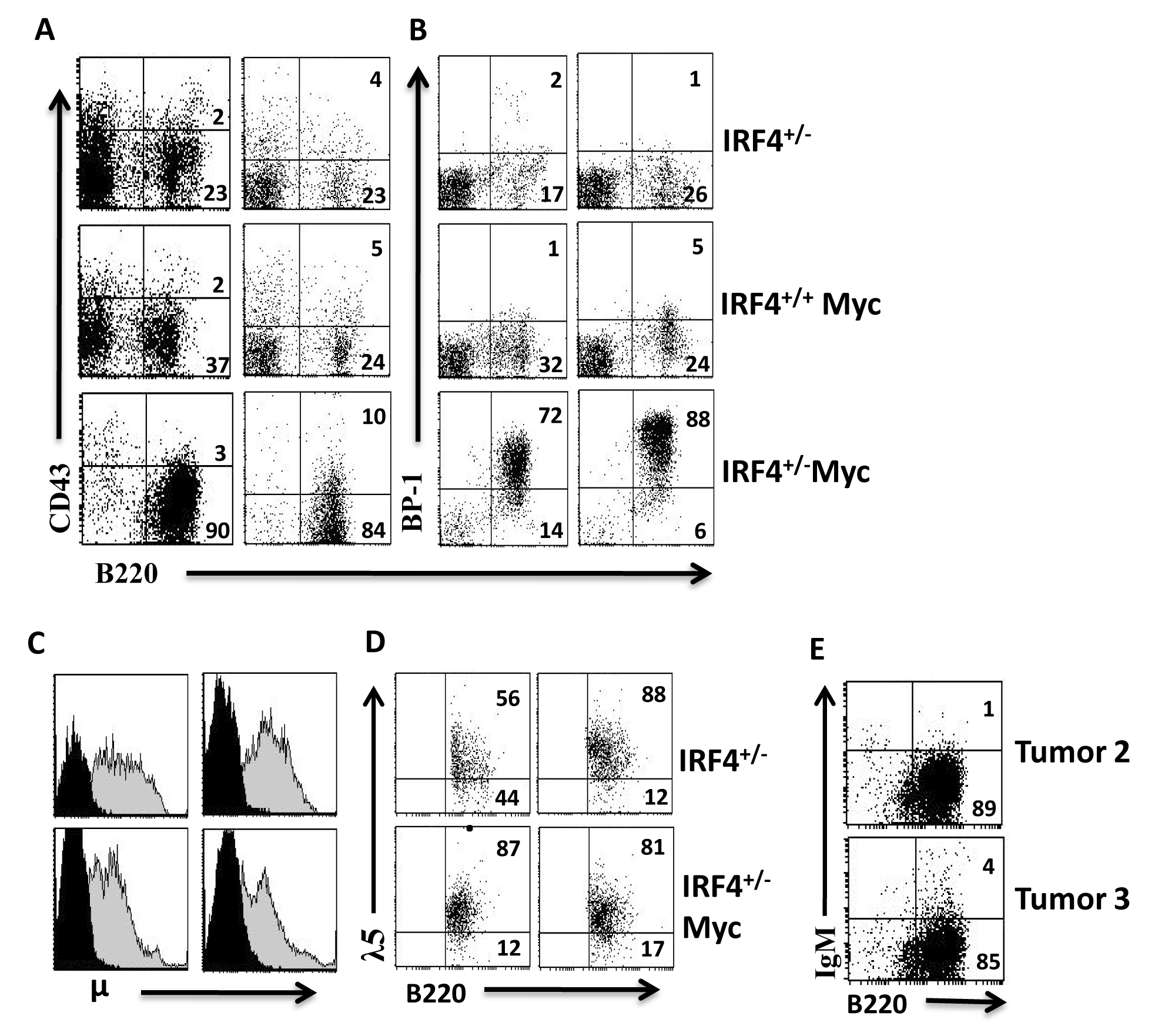

Supplement: Figure S2 — Two additional sets of independent experiments to show that IRF4+/−Myc leukemic cells were derived from large pre-B cells and were transplantable. A/B/) Bone marrow cells were isolated from six-week old IRF4+/−, IRF4+/+Myc and IRF4+/−Myc mice. The cells were stained with antibodies against CD43, Bp-1, CD19 and B220. The stained cells were analyzed by FACS. Numbers are percentages of cells in the respective quadrant. C) IRF4+/−Myc leukemic cells expressed intracellular μ. The bone marrow cells isolated above were stained with antibodies against B220 and CD43. After fixation and permeablization, the expression of intracellular heavy chain μ was detected with an anti-IgM antibody. The isotype IgG1 antibody staining was uses as a control for non-specific binding. The dark area (control IgG1) and light area (anti-IgM). D) IRF4+/−Myc cells expressed surrogate light chain λ5. Bone marrow cells isolated from IRF4+/− and IRF4+/−Myc mice were cultured in presence of IL-7 for two days. The expression of surrogate light chain λ5 was detected by FACS. E) Bone marrow cells were isolated from IRF4+/−Myc mice and transplanted into the non-irradiated syngenic host mice at 1×10^6^ cells per mice. A total of 10 host mice were used. The host mice were analyzed by FACS after 5 to 10 weeks. Two representative bone marrow analysis were shown. (TIF) [file pone.0022628.s002.tif]
